# *Melissa officinalis* Mitigates Psoriasis-Associated Cardiac Remodeling Through Antioxidant and Anti-Inflammatory Mechanisms

**DOI:** 10.3390/plants15152342

**Published:** 2026-07-30

**Authors:** Nevena Lazarevic, Marijana Andjic, Katarina Mihajlovic, Jovana Bradic, Aleksandar Kocovic, Djurdjina Petrovic, Dragana Stanisic, Vladimir Jakovljevic, Maja Muric, Jovana Joksimovic Jovic, Sergey Bolevich, Stefani Bolevich, Yakhyaev Huseyn, Vanja Tadic, Nenad Stankovic, Jasmina Sretenovic

**Affiliations:** 1 Department of Pharmacy, Faculty of Medical Sciences, University of Kragujevac, Svetozara Markovica 69, 34000 Kragujevac, Serbia; 2 Center of Excellence for Redox Balance Research in Cardiovascular and Metabolic Disorders, Svetozara Markovica 69, 34000 Kragujevac, Serbia; 3 Department of Human Pathology, 1st Moscow State Medical University IM Sechenov, Trubetskaya Street 8, Str. 2, 119991 Moscow, Russia; 4 Department of Dentistry, Faculty of Medical Sciences, University of Kragujevac, Svetozara Markovica 69, 34000 Kragujevac, Serbia; 5 Department of Physiology, Faculty of Medical Sciences, University of Kragujevac, Svetozara Markovica 69, 34000 Kragujevac, Serbia; 6 Department of Pathophysiology, 1st Moscow State Medical University IM Sechenov, Trubetskaya Street 8, Str. 2, 119991 Moscow, Russia; 7 Department of Pharmacology, 1st Moscow State Medical University IM Sechenov, Trubetskaya Street 8, Str. 2, 119991 Moscow, Russia; 8 Institute for Medicinal Plant Research Dr Josif Pancic, Tadeusa Koscuska 1, 11000 Belgrade, Serbia; 9 Independent Researcher, 11000 Belgrade, Serbia

**Keywords:** psoriasis, cardiac damage, *Melissa officinalis*, cardioprotection, oxidative stress, inflammation

## Abstract

Psoriasis is a chronic immune-mediated systemic disorder that extends beyond the skin and may adversely affect cardiovascular function. Herbal therapies have emerged as promising complementary approaches for mitigating psoriasis-associated cardiac complications. The present study aimed to evaluate the cardioprotective potential of *Melissa officinalis* (MO) extract in psoriasis. Twenty-four male Wistar albino rats were randomly assigned to four experimental groups: control, MO, psoriasis (PSORI), and PSORI+MO. After induction, animals received MO extract (200 mg/kg b.w.) orally for three weeks. Cardiac function was assessed using the Langendorff isolated heart preparation. Oxidative stress and inflammatory cytokines were determined in blood. Psoriatic rats exhibited impaired myocardial contractility and relaxation, accompanied by elevated SLVP and DLVP across all perfusion pressures. Administration of MO significantly improved cardiac performance and attenuated these hemodynamic disturbances. Oxidative stress markers and inflammatory cytokines were markedly reduced in the PSORI+MO group compared with PSORI. Cardiomyocyte diameter and cross-sectional area were increased in both PSORI and PSORI+MO groups relative to controls. Collagen content was substantially elevated in the PSORI group and remained increased, although to a lesser extent, in the PSORI+MO group compared with controls. These findings suggest that MO extract may represent a promising complementary strategy for reducing psoriasis-associated cardiovascular alterations.

## 1. Introduction

For decades, psoriasis has been recognized as a disease associated with systemic manifestations beyond the skin, including in the cardiovascular system. Until now it has been proven in clinical studies and meta-analyses that psoriatic patients, especially those with severe disease, frequently exhibit diastolic dysfunction, hypertrophy of the left ventricle, increased myocardial stiffness, and ECG changes such as prolonged QT interval, echocardiographic abnormalities, and atrial fibrillation even in the absence of proven overt cardiovascular disease [1,2]. Additionally, metabolic comorbidities associated with psoriasis, such as dyslipidemia, metabolic syndrome, obesity, diabetes, and insulin resistance, also contribute to the development of cardiovascular complications in these patients [3].

Several mechanisms have been proposed to describe how this cardiovascular damage in psoriasis actually happens. The central connection between psoriasis and psoriasis-associated cardiometabolic dysfunction is suggested to be systemic inflammation including the release of proinflammatory cytokines such as TNF-α, IL-17 and IL-23, and Th1/Th17 immune responses. This kind of inflammatory milieu contributes not solely to the skin, but also directly affects vascular inflammation, atherosclerotic plaque formation and instability, myocardial fibrosis, cardiomyocytes apoptosis and eventually cardiac dysfunction [1,4]. Besides chronic inflammation, another important mechanism connected with cardiovascular changes in psoriatic patients is oxidative stress. Overproduction of reactive oxygen species (ROS) leads to several consequences. It can damage the structure of biomolecules, including proteins, lipids, and DNA, induce damage and dysfunction of the mitochondria, and even amplify fibrotic changes leading to myocardial remodeling in psoriasis [5,6]. The interplay between systemic inflammation and oxidative stress has been singled out as the crucial pathogenetic mechanism of cardiovascular damage [1].

Thus, the role of natural products, especially plant extracts or isolated phytocomponents with strong antioxidant and anti-inflammatory potential, are increasingly being recognized in the search for novel therapeutic and preventive strategies in psoriatic patients [7].

Taking all of these into account, we hypothesized that *Melissa officinalis* (MO) extract may be beneficial in attenuating cardiovascular risk and reducing long-term cardiovascular complications in the psoriasis animal model. It is a well-known perennial herb for its health-promoting effects, traditionally called bee balm or lemon balm. Traditional use mostly involves treatment of palpitations, anxiety, insomnia, digestive problems, etc., but today, thanks to much evidence, its pharmacological effect on the cardiovascular system has been thoroughly investigated and demonstrated via several mechanisms including strong antioxidant potential (decrease of ROS production), anti-inflammatory (decreased production of proinflammatory cytokines) NF-κB activation, and antifibrotic action (prevention of myocardial remodeling) [8,9]. It is suggested that these protective effects of lemon balm are highly relevant to its bioactive constituents, including phenolic acids such as rosmarinic acid, chlorogenic, caffeic acids, etc., and flavonoid components such as luteolin, apigenin, etc. [9].

Our previous study demonstrated the beneficial effects of MO ethanolic extract on psoriatic skin changes after only one week of treatment, via reduction of proinflammatory markers and pro-oxidant production, together with antiproliferative action [10]. Building upon these findings, we wanted to investigate whether this extract could also attenuate cardiovascular alterations in psoriatic rats. The aim of the present study was to evaluate cardiodynamic and echocardiographic parameters of cardiac function, together with histological changes in the heart and systemic redox and inflammatory status analysis.

## 2. Results

### 2.1. Chemical Characterization of the Extract

**Figure 1:**
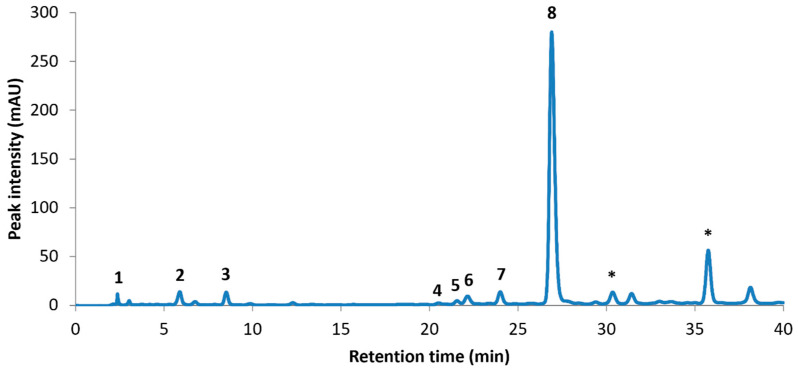
HPLC-DAD chromatogram of the *Melissa officinalis* extract. The numbered peaks correspond to the following compounds: (1) gallic acid, (2) chlorogenic acid, (3) caffeic acid, (4) rutin, (5) piceatannol, (6) luteolin-7-O-glucoside, (7) 3,5-di-O-caffeoylquinic acid, and (8) rosmarinic acid. The peak marked with an asterisk (*) corresponds to a p-hydroxycinnamic acid derivative (tentative identification). The corresponding UV absorption spectra are provided in the Supplementary Materials (Figures S1–S8).

HPLC fingerprint analysis revealed the presence of eight phenolic constituents of the analyzed MO extract (Figure 1, Table 1). It was shown that rosmarinic acid was the most abundant compound, accounting for 57.53 mg/g DW, whereas gallic acid, chlorogenic acid, and 3,5-di-O-caffeoylquinic acid were present in moderate amounts. On the other hand caffeic acid, stilbenoid piceatannol, flavonoids rutin, and luteolin-7-O-glucoside were detected at lower concentrations.

The chemical structures of the identified compounds are presented in Figure 2, while their corresponding UV absorption spectra are provided in the Supplementary Materials (Figures S1–S8).

### 2.2. In Vitro Antioxidant Activity

The total phenolic content (TPC) of MO was 75.43 ± 5.61 mg GAE/g dry extract. In the ferric reducing antioxidant power (FRAP) assay, MO showed a reducing power of 1.19 ± 0.21 µM Fe^2+^ equivalents, while ascorbic acid (AA) reached 2.31 ± 0.27 µM Fe^2+^ equivalents.

Significant differences in antioxidant capacity were observed among the tested samples in both assays (one-way ANOVA, *p* < 0.001). The extract exhibited significantly higher IC_50_ values compared with all reference antioxidants in both DPPH (2,2-diphenyl-1-picrylhydrazyl) and ABTS (2,2′-azino-bis-3-ethylbenzothiazoline-6-sulfonic) tests (Tukey post hoc, *p* < 0.001), indicating weaker radical-scavenging activity, while no statistically significant differences were detected among the standard antioxidants (AA, butylated hydroxyanisole (BHA), and Trolox) (Table 2).

### 2.3. Effects of the Administration of MO Extract on the Skin of Psoriatic Rats on

**Figure 3:**
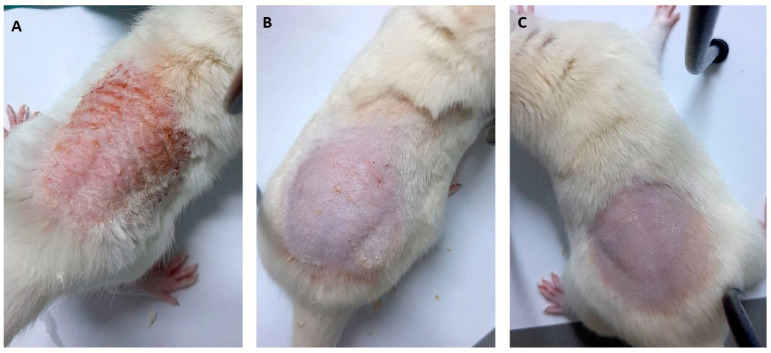
Macroscopic photos of psoriasis skin changes in rats: rat skin after induction of psoriasis (**A**), rat skin after 7 days of treatment with MO extract (**B**), rat skin after 14 days of treatment with MO extract (**C**).

Psoriasis induction resulted in severe erythema and desquamation of the dorsal skin (Figure 3A). Treatment with MO extract led to a gradual improvement of skin lesions, with reduced redness and scaling after seven days (Figure 3B) and near-complete resolution after fourteen days, when only minimal desquamation persisted (Figure 3C). Accordingly, the psoriasis area and severity index (PASI) score declined from 9 to 4 and finally to 1, demonstrating the therapeutic efficacy of the extract.

### 2.4. Body Weight (BW), Heart Weight (HW), and Index Hypertrophy of Heart (HW/BW Ratio)

In comparison to control values, the psoriasis (PSORI) and PSORI+MO groups showed increases in body weight of 16% and 17%, respectively. Both the PSORI and PSORI+MO groups had higher heart weights than the control (CTRL) and MO groups. In comparison to control values, the PSORI group’s index of hypertrophy of the heart (HW/BW ratio) increased by 14% and the PSORI+MO group’s by 8.6%. The heart weight/ body weight (HW/BW) ratio in psoriatic rats treated with MO was 7% lower than in the PSORI group (Table 3).

### 2.5. Echocardiographic Evauation

The echocardiographic analysis demonstrated that rats in the PSORI group exhibited significantly elevated LV internal dimension at end-diastole (LVIDd) values and reduced FS (fractional shortening) and EF (ejection fraction) compared with the CTRL and MO groups. Treatment with MO extract ameliorated these alterations, resulting in significantly lower LVIDd and LV internal dimension at end-systole (LVIDs) and higher FS and EF values when compared to untreated psoriatic animals (Table 4).

### 2.6. Cardiodynamic Parameters

**Figure 4:**
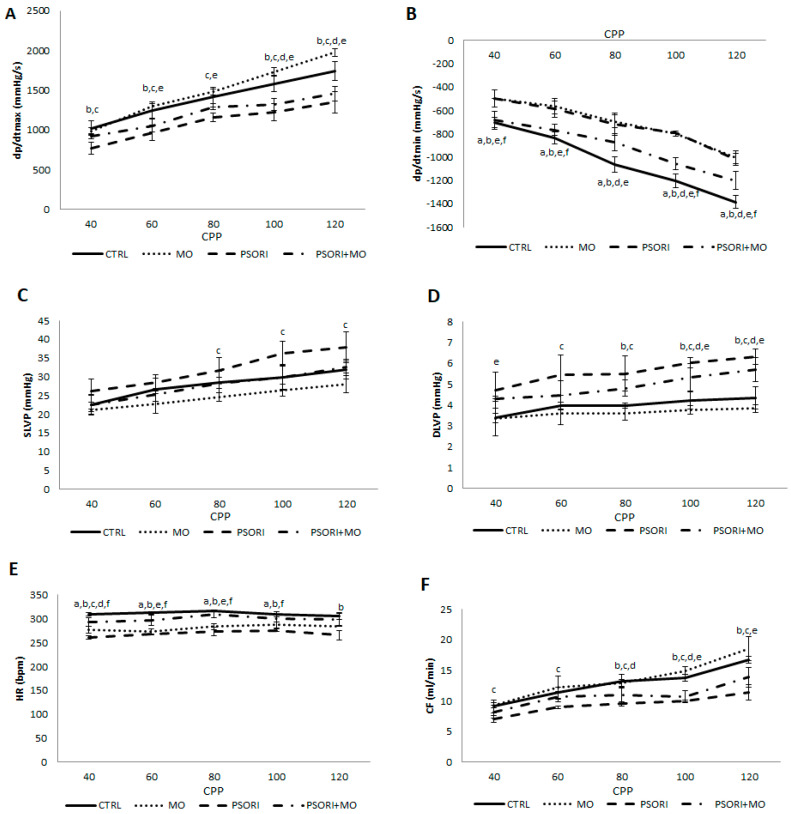
Effects of MO administration on the cardiodynamic parameters of the isolated rat heart. (**A**)—maximum rate of pressure development in the left ventricle; (**B**)—minimum rate of pressure development in the left ventricle; (**C**)—systolic left ventricular pressure; (**D**)—diastolic left ventricular pressure; (**E**)—heart rate; (**F**)—coronary flow. CTRL—healthy non-treated rats; MO—healthy rats treated with MO extract; PSORI—rats with psoriasis; PSORI+MO—rats with psoriasis treated with MO extract. Values are expressed as means ± STD. Statistical significance at the level of *p* < 0.05. a—MO vs. CTRL; b—PSORI vs. CTRL; c—PSORI vs. MO; d—PSORI+MO vs. CTRL; e—PSORI+MO vs. MO; f—PSORI+MO vs. PSORI.

The values of the maximum rate of pressure development in the left ventricle (dp/dt max) showed differences among the experimental groups across all examined coronary perfusion pressures (40–120 cm H_2_O). In the PSORI group, dp/dt max values were significantly decreased compared to the CTRL and MO groups at all perfusion pressures. In the MO group, dp/dt max values did not differ significantly from CTRL, but there was a statistically significant difference with the PSORI+MO group at CPP 60–120 cm H_2_O. In the group of rats with induced psoriasis treated with MO (PSORI+MO), dp/dt max values did not differ significantly from the PSORI group, while the values were statistically significantly decreased compared to the control group (Figure 4A).

The minimum rate of pressure development in the left ventricle (dp/dt min) was significantly increased in the PSORI group compared to CTRL at all coronary perfusion pressures, but there was no statistically significant difference in comparison with rats treated with MO extract. In the MO group, values were increased compared to the CTRL and PSORI+MO groups. In the PSORI+MO group, dp/dt min values were lower compared to the PSORI group, with statistically significant differences observed at almost all coronary perfusion pressures (except at 80 cm H_2_O). Also, there were increased dp/dt min values in the rats with psoriasis treated with MO extract in comparison with the control rats at CPP 80–120 cm H_2_O (Figure 4B).

Systolic left ventricular pressure (SLVP) values were only significantly increased in the PSORI group compared to MO at CPP 80–120 cm H_2_O, while we did not observe a statistically significant difference among other groups (Figure 4C).

Diastolic left ventricular pressure (DLVP) was significantly elevated in the PSORI group compared to the CTRL and MO groups at CPP 60–120 cm H_2_O. In the MO group, DLVP values were reduced compared to CTRL at higher perfusion pressures, reaching statistical significance at 100 and 120 cm H_2_O. In the PSORI+MO group, DLVP values were increased compared to the CTRL group, reaching statistical significance at CPP 100 and 120 cm H_2_O. Also, in this group, we observed elevated DLVP values compared to the MO group at CPP 40, 100, and 120 cm H_2_O (Figure 4D).

Heart rate (HR) values were decreased in almost all experimental groups compared to CTRL across the entire range of perfusion pressures (except in PSORI+MO at CPP 60–120 cm H_2_O). The decrease was most pronounced in the PSORI group, with statistically significant differences observed at all perfusion pressures. In the PSORI+MO group, HR values were significantly higher compared to the PSORI group at CPP 40–100 cm H_2_O. Interestingly, at CPP 120 cm H_2_O, there was only a statistically significant decrease in HR values in the PSORI group compared to the control group (Figure 4E).

The highest coronary flow (CF) values were observed in the MO group, with statistically significant differences compared to PSORI at all perfusion pressures. Statistically significant decreases were observed in the PSORI group compared to CTRL at perfusion pressures starting from 80 to 120 cm H_2_O. In the PSORI+MO group, there were statistically significantly lower CF values compared to CTRL at perfusion pressures 80 and 100 cm H_2_O, while these values were decreased in comparison with the MO group at 100–120 cm H_2_O (Figure 4F).

### 2.7. Redox Status

**Figure 5:**
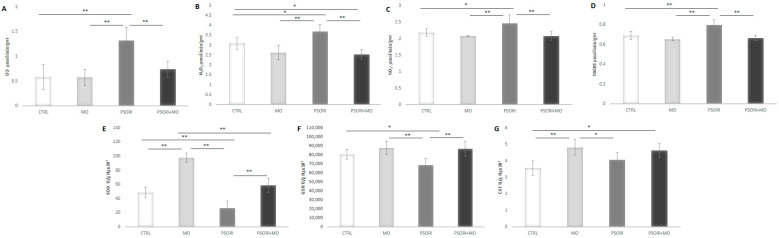
Effects of MO on the markers of oxidative stress. (**A**)—superoxide anion radical O_2_^−^; (**B**)—hydrogen peroxide (H_2_O_2_); (**C**)—nitrites (NO_2_^−^); (**D**)—index of lipid peroxidation measured as TBARS; (**E**)—superoxide dismutase (SOD); (**F**)—reduced glutathione (GSH) and (**G**)—catalase (CAT). CTRL—healthy non-treated rats; MO—healthy rats treated with MO extract; PSORI—rats with psoriasis; PSORI+MO—rats with psoriasis treated with MO extract. Values are expressed as means ± STD. *—statistical significance at the level of *p* < 0.05 between marked groups, ** — statistical significance at the level of *p* ˂ 0.005 between marked groups.

Pro-oxidative markers measured in plasma are represented in Figure 5A–D, while antioxidant defense markers measured from lysate are represented in Figure 5E–G. Psoriasis induction led to a significant increase of all measured pro-oxidant markers compared to healthy CTRL rats (Figure 5A–D). Administration of MO significantly reduced superoxide anion radical (O_2_^−^) levels in psoriatic animals, although values remained slightly higher than in controls. Similarly, hydrogen peroxide (H_2_O_2_) concentrations were significantly increased in the PSORI group, while treatment markedly decreased H_2_O_2_ production compared to untreated psoriatic rats (Figure 5B). NO_2_^−^ levels, used as an indirect indicator of nitric oxide production, were also significantly elevated in the PSORI group relative to CTRL and MO animals (Figure 5C). Supplementation with lemon balm extract significantly attenuated this increase, restoring nitrite (NO_2_^−^) values close to those observed in controls. A similar trend was observed for thiobarbituric acid reactive substances (TBARS), where lipid peroxidation was markedly enhanced in the PSORI group and significantly reduced following treatment (Figure 5D).

Regarding antioxidant parameters, induction of psoriasis significantly decreased superoxide dismutase (SOD) activity and reduced glutathione (GSH) levels compared to healthy animals (Figure 5E,F), while MO treatment successfully restored these values compared to PSORI+MO (Figure 5E,F), similar to the ones in the CTRL group (Figure 5E,F). On the contrary, catalase (CAT) activity was not marked among experimental groups with psoriasis, although an increase was observed in MO-treated animals compared to CTRL animals (Figure 5G).

### 2.8. Inflammatory Status

**Figure 6:**
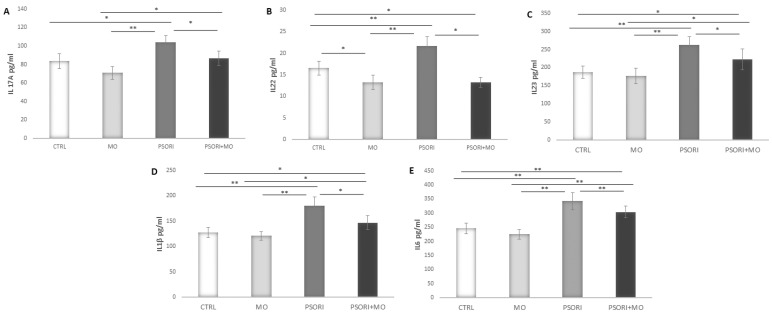
Effects of MO on the serum cytokine levels in the psoriasis model. (**A**)—IL-17A; (**B**)—IL-22; (**C**)—IL-23; (**D**)—IL-1beta; (**E**)—IL-6. CTRL—healthy non-treated rats; MO—healthy rats treated with MO extract; PSORI—rats with psoriasis; PSORI+MO—rats with psoriasis treated with MO extract. Values are expressed as means ±STD. *—statistical significance at the level of *p* < 0.05 between marked groups, **—statistical significance at the level of *p* ˂ 0.005 between marked groups.

Psoriatic animals exhibited a significant increase in all measured proinflammatory markers compared to the CTRL group of healthy rats, including IL-17A, IL-22, IL-23, IL-1β, and IL-6 (Figure 5A–E). Among the analyzed cytokines, IL-6 demonstrated the most prominent elevation in the PSORI group. Administration of MO significantly reduced IL-6 concentrations compared to untreated psoriatic rats; however, levels remained higher than those observed in the CTRL and MO groups (Figure 6E). Three-week treatment with MO attenuated the psoriatic inflammatory response, as seen by significant reduction of all measured cytokine concentrations in the PSORI+MO group compared to untreated animals with psoriasis (Figure 6A–E). The most pronounced effect of MO was observed in IL-17A level, since the treatment successfully lowered these values, similar to control ones (Figure 6A).

### 2.9. Morphometry

**Figure 7:**
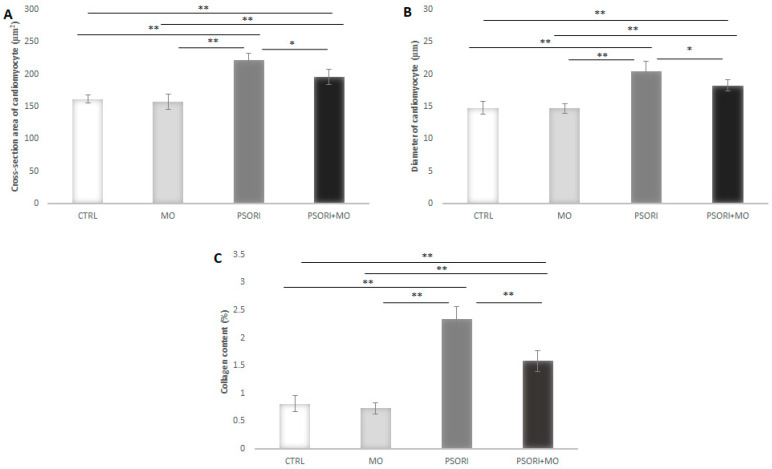
Morphometric analysis of the heart: (**A**)—cross-sectional area of cardiomyocytes; (**B**)—diameter of cardiomyocytes; (**C**)—collagen content in the heart. CTRL—healthy non-treated rats; MO—healthy rats treated with MO extract; PSORI—rats with psoriasis; PSORI+MO—rats with psoriasis treated with MO extract. Values are expressed as means ± SD. *—statistical significance at the level of *p* < 0.05 between marked groups, **—statistical significance at the level of *p* ˂ 0.005 between marked groups.

The cross-sectional area (CSA) of cardiomyocytes increased by 37% in the PSORI group and by 21% in the PSORI+MO group relative to control animals. Treatment with an MO extract in psoriatic rats reduced the cardiomyocyte CSA by 16% compared with the PSORI group (Figure 7A). Similarly, the longitudinal diameter of cardiomyocytes was elevated by 39% in the PSORI group and by 23% in the PSORI+MO group compared to controls. In comparison with psoriasis alone, administration of MO extract significantly lowered cardiomyocyte diameter by 11% in psoriatic rats (Figure 7B). Cardiac collagen content was markedly increased in the PSORI group (187%) and in the PSORI+MO group (94%), whereas the MO group showed a 10% reduction compared to control values. Moreover, collagen content in the PSORI+MO group was reduced by 33% relative to the PSORI group (Figure 7C).

### 2.10. Histopathological Analyses of the Myocardium

**Figure 8:**
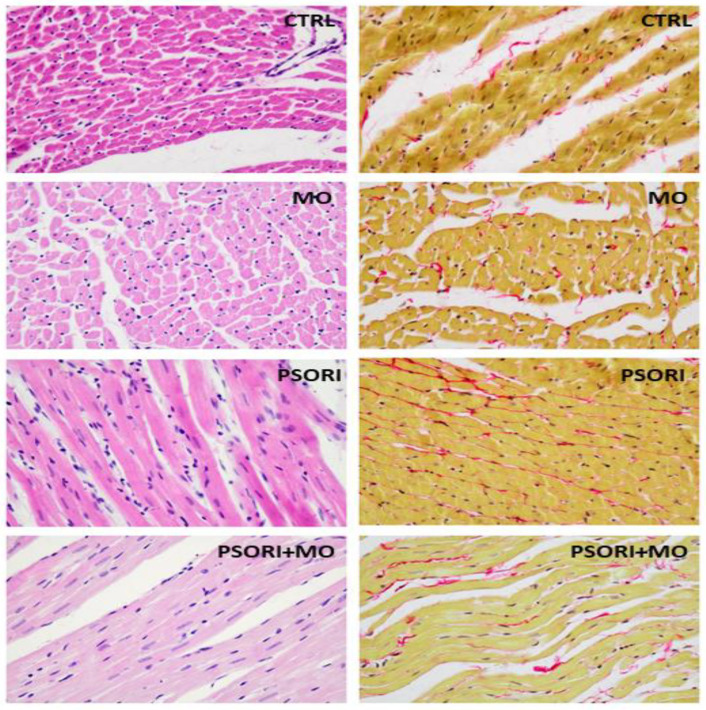
Histological analysis of the heart: hematoxylin eosin staining (**left**) and sirius red staining (**right**). Magnification 200×. CTRL—healthy non-treated rats; MO—healthy rats treated with MO extract; PSORI—rats with psoriasis; PSORI+MO—rats with psoriasis treated with MO extract.

Microphotographs of hematoxylin and eosin (H/E)-stained cardiac tissue (Figure 8, left panel) from the CTRL and MO groups revealed preserved myocardial architecture without evidence of inflammation or cardiomyocyte hypertrophy. In contrast, the PSORI group exhibited disrupted tissue morphology characterized by inflammatory infiltration, cardiomyocyte hypertrophy, and widening of the interstitial space. Treatment with MO extract markedly attenuated these psoriasis-associated histopathological alterations. In the PSORI+MO group, cardiac tissue displayed largely preserved morphology with no detectable inflammation, although mild cardiomyocyte hypertrophy remained present. Picrosirius red-stained sections of cardiac tissue (Figure 8, right panel) demonstrated collagen fiber deposition appearing as red-stained areas. The PSORI group showed more pronounced, coarse, and densely bundled collagen deposits. Administration of MO extract to psoriatic rats reduced collagen accumulation in the myocardium, which was further confirmed by image analysis.

## 3. Discussion

Psoriasis is a chronic immune-mediated inflammatory skin disorder that is increasingly recognized as a systemic disease rather than a condition limited to the skin. In addition to its cutaneous manifestations, accumulating evidence suggests that psoriasis is associated with systemic complications, particularly an increased risk of cardiovascular dysfunction, which may be partly related to the severity and duration of the disease. However, the mechanistic link between skin inflammation and cardiovascular alterations remains incompletely understood, and experimental evidence regarding potential therapeutic strategies targeting both local and systemic effects are still limited [10].

In this context, plant-derived bioactive compounds have gained increasing attention due to their multitarget pharmacological properties, including anti-inflammatory, antioxidant, and immunomodulatory activities. MO is a well-known medicinal plant traditionally used for its calming, anti-inflammatory, and skin-protective effects. Previous studies have demonstrated that its phenolic constituents may exert beneficial effects on oxidative stress-related pathways and inflammatory responses, suggesting their potential relevance in complex inflammatory conditions such as psoriasis [9,11].

Based on the above, the present study was designed to evaluate the systemic effects of MO extract in an experimental model of psoriasis in rats, with a particular focus on both cutaneous alterations and potential cardiovascular implications.

In the first phase of the present study, a detailed HPLC analysis was performed to characterize the phytochemical composition of the MO extract and identify its principal phenolic constituents. The HPLC profile obtained in the present study confirmed that rosmarinic acid is the predominant phenolic constituent of MO extract, which is in strong agreement with previous phytochemical investigations reporting this compound as the major marker of lemon balm. In hydroalcoholic extracts of MO, rosmarinic acid has been consistently described as the most abundant phenolic, with reported concentrations showing marked variability depending on extraction solvent and plant origin, but generally representing the dominant fraction of total phenolics [8,12].

For instance, Wojdylo et al. reported that rosmarinic acid was the principal compound in lemon balm leaves, substantially exceeding the levels of other phenolic acids, while other studies on ethanolic extracts similarly identified chlorogenic and caffeic acid derivatives as secondary constituents present in moderate amounts [13]. The relative abundance pattern observed in the present extract, characterized by a clear predominance of rosmarinic acid followed by chlorogenic and caffeic acid derivatives, therefore aligns well with previously reported chromatographic profiles of this species [8,11]. In contrast, some studies have reported lower proportions of rosmarinic acid or a more balanced distribution of phenolic acids, which has been attributed to differences in extraction methodology, geographical origin, harvesting time, and post-harvest processing [11]. The presence of flavonoids such as rutin and luteolin-7-O-glucoside at lower concentrations is also consistent with literature data, where these compounds are typically reported as minor constituents in MO compared to phenolic acids [14].

Following the phytochemical characterization, the TPC and antioxidant potential of the MO extract were evaluated. The obtained TPC confirms that the MO extract contains a high level of phenolic compounds, although considerable variability in TPC values has been reported in the literature, mainly due to differences in plant origin and extraction procedures. Previous studies have reported TPC values ranging from approximately 30 to 150 mg GAE/g extract, placing the value obtained in the present study within the range commonly reported for hydroalcoholic and ethanolic MO extracts [8,14].

The antioxidant assays confirmed the ability of the extract to scavenge free radicals and reduce ferric ions, although its activity was lower than that of the reference antioxidants. This observation is expected, as Trolox, ascorbic acid, and BHA are pure compounds with high antioxidant potency, whereas plant extracts contain complex mixtures of bioactive constituents at lower concentrations. Similar findings have been reported for MO extracts in previous studies. The observed antioxidant activity is likely associated with the predominance of rosmarinic acid, together with other phenolic acids and flavonoids identified in the extract [12,13]. Given the well-established role of oxidative stress in psoriasis pathogenesis, the demonstrated antioxidant capacity of the investigated extract provides a plausible basis for its further evaluation in vivo and its potential protective effects against psoriasis-associated tissue damage.

Following the promising phytochemical profile and antioxidant activity observed in vitro, the second phase of the study was focused on the evaluation of the biological effects of MO extract in an experimental model of psoriasis. Given the well-established role of oxidative stress and systemic inflammation in the pathogenesis of psoriasis and its associated cardiovascular comorbidities, the in vivo investigation was designed to assess the potential cardioprotective effects of chronic systemic administration of MO extract in psoriatic rats.

In addition to evaluating local skin alterations, particular attention was directed toward cardiovascular function and oxidative stress parameters, considering the growing recognition of psoriasis as a systemic inflammatory disorder associated with an increased risk of cardiovascular complications. This approach enabled the assessment of whether the antioxidant and anti-inflammatory potential demonstrated in vitro could translate into beneficial effects in vivo, both at the site of inflammation and in distant organs affected by psoriasis-associated systemic changes.

Consistent with the antioxidant potential demonstrated in vitro, systemic administration of MO extract exerted pronounced effects on oxidative stress status in vivo. Psoriasis induction resulted in a marked disturbance of systemic redox homeostasis, as evidenced by elevated levels of pro-oxidative markers and impaired antioxidant defense mechanisms. These findings are in accordance with previous experimental and clinical studies demonstrating increased production of reactive oxygen and nitrogen species, enhanced lipid peroxidation, and depletion of endogenous antioxidants in psoriasis, all of which contribute to disease progression and the development of systemic complications [15,16]. In the present study, treatment with MO significantly attenuated the psoriasis-induced increase in O_2_^−^, H_2_O_2_, NO_2_^−^, and TBARS levels, indicating a reduction in oxidative damage and lipid peroxidation. Similar antioxidant effects of MO have been reported in other experimental models characterized by oxidative stress, where supplementation with lemon balm extracts reduced reactive oxygen species generation and improved overall redox balance. These effects have largely been attributed to the high content of rosmarinic acid and other phenolic constituents, which are capable of scavenging free radicals and suppressing oxidative chain reactions [14]. The beneficial effects of MO were further supported by restoration of endogenous antioxidant defenses. The observed increase in SOD activity and GSH levels following treatment is consistent with previous reports showing that MO and its major phenolic constituents can enhance antioxidant enzyme activity and preserve intracellular glutathione stores under conditions of oxidative stress. Similar findings have been reported in models of hepatic injury, neurotoxicity, and metabolic disorders, where administration of MO extracts significantly improved antioxidant status and reduced oxidative damage. In contrast, CAT activity was not substantially affected by psoriasis induction, suggesting that the SOD–GSH axis may represent the primary antioxidant defense mechanism influenced by MO treatment in the present model. The observed modulation of oxidative stress markers is particularly relevant in psoriasis, as oxidative imbalance is considered a key contributor not only to keratinocyte hyperproliferation and inflammation but also to psoriasis-associated cardiovascular dysfunction. Therefore, restoration of systemic redox homeostasis may represent one of the principal mechanisms through which MO exerts its protective effects in psoriatic animals [17,18].

What is more, besides oxidative stress reduction and inflammation attenuation, the applied MO extract was shown to induce significant improvement in the structure of the myocardium. Both histological and morphometric analysis revealed decreased inflammatory deposition, collagen content and cardiomyocyte size after 3-week treatment with MO extract in psoriatic animals. Chronic inflammatory diseases, including psoriasis, are often associated with fibrosis of the myocardium, leading to remodeling of the heart and subsequent impaired performance of the heart. We confirmed this finding in our study, while the MO treatment reversed these effects and preserved myocardial structure. Different mechanisms of fibrosis promotion have been described in the literature, including proinflammatory cytokines, especially IL-17 and IL-6 overlapping with overproduction of ROS [19,20]. Considering the reductions in IL-17A and IL-6 levels together with the attenuation of oxidative stress observed in our study, it is plausible that the antifibrotic effects of MO result from the synergistic interplay between its anti-inflammatory and antioxidant activities. Antifibrotic properties of MO were also confirmed in other studies involving inflammatory heart disease such as autoimmune myocarditis [21]. Additionally, the Lamiaceae family of plants, which contain rosmarinic acid, have been proved to exert strong antifibrotic action on various organs, including the heart, via different mechanisms such as suppression of profibrotic TGF-β and Wnt. signaling pathways, and activation of protective pathways such as PPARγ, AMPK, and NRF2 [22]. Since rosmarinic acid was also found to be major component of our extract also, it can be considered responsible for achieved antifibrotic effects on psoriatic rat hearts in synergy with other isolated phenolics and flavonoids.

The functional analyses of the heart we conducted in this study supported the structural histological and morphometric findings. Namely, the echocardiographic changes found were modest including the improvement of LVIDd and LVIDs, EF and FS after MO treatment, indicating that MO may preserve cardiac function in the early stage of psoriasis-induced heart injury, prior to the development of pronounced structural remodeling. Cardiodynamic assessment using the Langendorff model of an isolated rat heart revealed that psoriasis induction significantly impaired myocardial performance, as proved by reduced contractility (lower dp/dt max), impaired relaxation (higher dp/dt min and DLVP), reduced heart rate, and diminished coronary flow. The present study revealed that psoriasis induced significant structural and functional alterations in the heart. Cardiac contractility and relaxation were assessed by measuring dp/dt max and dp/dt min, respectively. The disease markedly impaired myocardial contractile performance, as indicated by reduced dp/dt max values across all perfusion pressures. Likewise, cardiac relaxation was adversely affected, with lower dp/dt min values observed at higher perfusion pressures. In contrast, treatment with MO effectively attenuated these detrimental effects and improved myocardial contractility. These findings are consistent with previous studies demonstrating the cardioprotective potential of MO, including our earlier work, in which administration of the extract enhanced the inotropic function of the heart following ischemia–reperfusion injury. Similar findings were confirmed in previous research suggesting that chronic systemic inflammation and oxidative stress contribute to myocardial dysfunction and microvascular impairment in psoriasis [23].

Previous studies have reported an association between psoriasis and hypertension, although the underlying mechanisms remain incompletely understood [23,24]. Both angiotensin II and oxidative stress are thought to contribute to this relationship by promoting inflammation, vascular dysfunction, and adverse cardiovascular remodeling. In the present study, psoriatic rats exhibited elevated systolic and diastolic left ventricular pressures across all perfusion pressures. Such hemodynamic changes may contribute to cardiac hypertrophy, which was supported by our echocardiographic and morphometric findings showing structural alterations of the myocardium in the PSORI group.

In addition, impaired cardiac relaxation was observed in psoriatic animals, resulting in increased diastolic left ventricular pressure. Reduced ventricular compliance may limit diastolic filling and subsequently compromise systolic performance. Although the exact mechanisms remain unclear, myocardial hypertrophy and increased collagen deposition, both detected in the PSORI group, likely contributed to the observed diastolic dysfunction. Enhanced oxidative stress may represent another important factor involved in these alterations.

Furthermore, adipose tissue is considered a major source of angiotensinogen [23,24] in psoriasis. Since the greatest increase in body weight was observed in the PSORI group, it is plausible that angiotensin II production was also elevated, potentially contributing to the cardiovascular abnormalities identified in this study. Treatment with MO in psoriatic rats improved SLVP and DLVP values. These results were in accordance with our previous findings that MO extract improved the heart’s systolic and diastolic functions [25].

Even though MO treatment did not achieve complete normalization of all cardiodynamic parameters measured, it induced a partial recovery of the heart via reducing diastolic dysfunction and improving heart rate and CF drop, suggesting MO’s ability to preserve cardiac performance and coronary circulation. Similar findings were found in other papers investigating the influence of MO on isolated rat hearts [25,26].

The ability of the disease to promote cardiac hypertrophy was demonstrated through the assessment of the cardiac hypertrophy index and measurements of cardiomyocyte diameter and cross-sectional area, in agreement with previous findings [23]. Morphometric evaluation revealed that psoriasis markedly increased cardiomyocyte size, as evidenced by enlarged cell diameter and cross-sectional area. This result is confirmed in the study of Corbic and colleagues [23]. Furthermore, the elevated cardiac hypertrophy index (HW/BW ratio) observed in psoriatic rats was consistent with both morphometric indicators of myocardial hypertrophy. The obtained results also indicated that psoriasis contributed to the development of cardiac fibrosis. Collectively, these findings suggest that the disease induces structural remodeling of the myocardium and adversely affects cardiac function, which is also confirmed in the previous study [23]. Treatment with MO extract significantly alleviated cardiac remodeling in psoriatic rats, as evidenced by reduced cardiomyocyte hypertrophy and lower collagen accumulation in the myocardium. The observed cardioprotective effects are likely related to the high content of rosmarinic acid and gallic acid, the major polyphenolic constituents of the extract, which may contribute to the attenuation of myocardial hypertrophy and fibrosis.

On the other hand, rosmarinic acid may be primarily responsible for the observed effects. Previous studies have demonstrated that oral administration of isolated rosmarinic acid exerts antifibrotic effects and reduces systolic left ventricular pressure (SLVP), as well as the maximum and minimum rates of left ventricular pressure development (dp/dt max and dp/dt min), in rats with experimentally induced myocardial infarction. These antifibrotic effects are attributed to a reduction in the collagen volume fraction through the downregulation of collagen type I, collagen type III, hydroxyproline, and α-smooth muscle actin (α-SMA) expression. Since collagen deposition was significantly reduced following *Melissa officinalis* extract treatment in our study, we hypothesize that rosmarinic acid may have contributed to this effect through a similar mechanism and could therefore be largely responsible for the attenuation of cardiac fibrosis. Furthermore, rosmarinic acid has been shown to decrease the expression of angiotensin-converting enzyme (ACE) and angiotensin II type 1 receptor (AT1R), while increasing the expression of angiotensin-converting enzyme 2 (ACE2), thereby enhancing cardioprotective effects against cardiac dysfunction and fibrosis [22]. Since *Melissa officinalis* treatment in our study also reduced both systolic and diastolic left ventricular pressure, we speculate that these cardioprotective effects may have been mediated, at least in part, through the same molecular mechanism. However, since the whole extract was not directly compared with pure rosmarinic acid, its individual contribution cannot be established, and additive or synergistic effects of other phenolic constituents cannot be excluded. This represents a limitation of the present study and should be addressed in future investigations.

Moreover, an important consideration when interpreting these findings is the oral bioavailability of the polyphenolic constituents of MO, particularly rosmarinic acid, the predominant compound identified in the extract. Although rosmarinic acid undergoes extensive gut microbial and phase II metabolism after oral administration, both the parent compound and its metabolites reach the systemic circulation and have been associated with biological activity following repeated dosing [27,28]. Therefore, rosmarinic acid, together with other phenolic constituents, likely contributed to the antioxidant and anti-inflammatory effects observed in this study. However, plasma or tissue concentrations of these compounds were not determined and should be evaluated in future pharmacokinetic studies. Current therapies for psoriasis, including biological therapy oriented towards TNF-α, IL-17, IL-22 and IL-23, remains efficient in terms of improving skin changes and possible cardiovascular consequences, but is associated with adverse effects and economic burden. So, the interest in plant extracts as adjuvant therapies is justified, since they are generally considered a safer and more cost-effective option. In that sense, these findings provide scientific evidence that supports the potential use of MO extract as an adjuvant therapeutic approach in psoriatic patients.

## 4. Materials and Methods

### 4.1. Plant Material and Extract Preparation

Leaves of *Melissa officinalis* (MO) were used for the preparation of the plant extract. A total of 100 g of dried leaves was purchased from Bilje Borca (Belgrade, Serbia), pulverized using an IKA A11 analytical mill (Staufen im Breisgau, Germany), and kept at room temperature until extraction. The extraction procedure was performed on the powdered dried leaves by reflux extraction using 70% ethanol as the extraction solvent for 2.5 h. Following extraction, the obtained mixture was initially filtered through gauze, while the filtrate was left to stand at ambient temperature to allow sedimentation of insoluble ballast substances. The clarified supernatant was subsequently passed through Whatman No. 1 filter paper. Ethanol was removed under reduced pressure employing a rotary evaporator (RV05 Basic IKA, Staufen im Breisgau, Germany) operated at 40 °C, 90 rpm, and 250 mbar, yielding a dry extract. The dry extract was stored at 4 °C until analysis [14].

### 4.2. Chemical Characterization of the Obtained Extract

The HPLC fingerprint of the investigated MO extract was obtained using an Agilent Technologies 1200 HPLC system equipped with a diode array detector (DAD). Chromatographic data were recorded at a wavelength of 320 nm. Separation of constituents was performed on a LiChrospher 100 RP-18e (5 μm, 250 × 4 mm i.d.) column at a flow rate of 0.8 mL/min. The mobile phase consisted of solvent A (0.1 M phosphoric acid, H_3_PO_4_) and solvent B (acetonitrile, MeCN), applied under gradient elution conditions as follows: 89–75% A (0–20 min), 75–60% A (20–35 min), and 60–35% A (35–40 min). Prior to analysis, the sample solution (3.01 mg/mL in 70% ethanol; dry weight = 4.1%), prepared as previously described, was filtered through 0.45 μm PTFE membrane filters (Fisher Scientific, Pittsburgh, PA, USA). A 4 μL aliquot was injected for analysis. Standard solutions used for the identification of polyphenolic compounds were prepared in ethanol at the following concentrations: 0.58 mg/mL for protocatechuic acid, 0.34 mg/mL for luteolin-7-O-glucoside, 0.39 mg/mL for 3,5-di-O-caffeoylquinic acid, 0.52 mg/mL for caffeic acid, 0.30 mg/mL for neochlorogenic acid, and 0.34 mg/mL for rosmarinic acid. A reference library of phenolic standards was constructed by individual injections of each compound under identical chromatographic conditions, with detection performed using a photodiode array detector. Compound identification was based on comparison of retention times and UV–Vis spectral characteristics. After spectral matching, additional confirmation was achieved by co-injection (spiking) with authentic standards, followed by peak purity assessment using a three-point purity test and spectral similarity analysis against the reference library. Peaks showing high spectral concordance and matching retention times with standards were considered positively identified, while those not meeting these criteria were excluded from quantification. The method showed good reproducibility, with relative standard deviations for retention times ranging from 0.18 to 1.79% across three consecutive runs. Quantification was performed using external calibration curves constructed from authentic standards. All results are presented as mean ± standard deviation of three independent measurements.

### 4.3. In Vitro Antioxidant Activity of the Obtained Extract

TPC was determined using the Folin–Ciocalteu colorimetric method [29]. Briefly, 100 μL of ethanolic extract solution was mixed with 0.75 mL of 10-fold diluted Folin–Ciocalteu reagent and incubated for 5 min at 22 °C. Subsequently, 0.75 mL of sodium bicarbonate solution (60 g/L) was added. After 90 min of incubation at room temperature, absorbance was recorded at 725 nm. Gallic acid solutions (0–100 mg/L) were used for preparation of the calibration curve. The TPC was expressed as mg GAE per g of dry weight. All measurements were performed in triplicate, and the results are presented as mean ± SD.

The antioxidant activity of the samples was assessed using a modified microplate-based DPPH radical scavenging assay adapted for high-throughput analysis [12]. Briefly, a 0.004% (*w*/*v*) methanolic DPPH solution was mixed with different concentrations of the investigated samples or reference antioxidants in 96-well microplates to obtain a final reaction volume of 200 μL per well. The reaction mixtures were incubated for 30 min at room temperature in the dark, after which absorbance was measured at 515 nm using a microplate reader. Antioxidant activity was expressed as the percentage of DPPH radical inhibition relative to the control sample containing only DPPH solution. In addition, IC_50_ values, representing the concentration required to inhibit 50% of DPPH radicals, were calculated for all tested samples. Assay reliability was confirmed using standard antioxidant compounds analyzed under the same experimental conditions.

Antioxidant capacity was additionally determined using the ABTS radical cation decolorization assay [12]. The ABTS^+^∙ radical solution was generated by reacting ABTS stock solution with potassium persulfate and keeping the mixture in the dark for 16 h at room temperature. Before analysis, the solution was diluted with ethanol to obtain an absorbance of 0.700 ± 0.020 at 734 nm. For the assay, 20 μL of the tested sample or reference antioxidant was mixed with 180 μL of ABTS^+^∙ working solution in a 96-well microplate. The reduction in absorbance was measured at 734 nm within 1 min after mixing. All measurements were performed in triplicate, and the results are presented as mean values ± SD.

The FRAP assay was used to evaluate the electron-donating ability of the investigated extracts [22]. The FRAP reagent was freshly prepared by mixing 300 mM acetate buffer (pH 3.6), 10 mM TPTZ solution in 40 mM HCl, and 20 mM FeCl_3_ solution in a 10:1:1 (*v*/*v*/*v*) ratio. For analysis, 10 μL of sample was added to 300 μL of FRAP reagent in a microplate well. The reaction mixture was incubated at 37 °C for 10 min, allowing reduction of the Fe^3+^-TPTZ complex to the ferrous form. Absorbance was then measured at 593 nm, using distilled water as blank. Trolox was used as the reference antioxidant, and calibration was performed with ferrous sulfate standards. Results were expressed as μmol Fe^2+^ equivalents.

### 4.4. Ethical Considerations

All animal experiments conducted in this study were reviewed and approved by the Ethics Committee for the Welfare of Experimental Animals at the Faculty of Medical Sciences, University of Kragujevac (Kragujevac, Serbia), under approval No. 01-10948/5, approval date 10 October 2022. The procedures adhered to the guidelines set forth in the European Directive 2010/63/EU on the protection of animals used for scientific purposes, complied with the principles of good laboratory practice (GLP), and followed the provisions of the earlier Directive 86/609/EEC concerning the care and use of laboratory animals.

### 4.5. Animals

For the purpose of the study, we used Wistar albino male rats (*Rattus norvegicus*)*,* 10 weeks old, weighing 250 ± 20 g, a total of 24 rats. Rats were obtained from the Animal House of the Military Medical Academy (Belgrade, Serbia). The animals were kept under standardized, controlled environmental conditions (room temperature 21 ± 2 °C, humidity 55 ± 5%, and 12 h dark–light cycle) with ad libitum access to standard rat food (standard laboratory diet composed of 9% fat, 20% protein, and 53% carbohydrates) and drinking water. Animals were monitored daily for general health status, behavior, and signs of distress throughout the experimental period.

In order to minimize selection bias, after 1 week of adaptation to the new environment, the rats were randomly divided into 4 experimental groups (6 rats per group) and housed in polyethylene cages (1 rat per cage):

1. CTRL—control group of healthy animals (n = 6),
2. MO—healthy rats treated with systemic administration of *Melissa officinalis* extract (n = 6),
3. PSORI—rats with induced psoriasis (n = 6),
4. PSORI+MO—rats with induced psoriasis treated with systemic administration of *Melissa officinalis* extract (n = 6).

### 4.6. Study Protocol

A well-established animal model of psoriasis was used in the study. That is, the disease was induced by topical application of imiquimod cream (^®^Aldara, 50 mg of 5% imiquimod) to the shaved back skin of rats, once a day for one week (7 consecutive days). The disease induction was confirmed by using PASI. Erythema, scaling, and skin thickness were evaluated and scored independently on a scale ranging from 0 to 4 (0 = none; 1 = mild; 2 = moderate; 3 = marked; 4 = very marked), yielding a maximum cumulative PASI score of 12 [17]. Rats belonging to MO and PSORI+MO were administered ethanolic MO extract dissolved in tap water *per os* (*ex tempore*, prior to application), every day at the same time for 3 weeks in a dose of 200 mg/kg [18]. The selected dose of МО extract (200 mg/kg b.w.) was chosen based on previously published pharmacological studies demonstrating its biological efficacy and safety in experimental animal models [14,18].

### 4.7. Echocardiographic Measurements

After finishing the protocol (on the 28th day of the experiment), prior to scarification, animals were subjected to transthoracic echocardiography. Rats were anesthetized with isoflurane (RWD Life Science Co., Ltd. Shenzhen, China) and examined with the Hewlett Packard Sonos 5500 ultrasound system (Andover, MA, USA) with 15.0 MHz phased-array transducer. M-mode images were obtained from the parasternal long-axis view, with measurements, averaged over minimum 5 cardiac cycles, taken perpendicular to the inter-ventricular septum (IVS) and LV posterior wall (LVPW) at the papillary muscle level. Left ventricle dimensions were measured: IVS wall thickness at end-diastole (IVSd) and end-systole (IVSs), LVIDd and LVIDs, LVPW thickness at end-diastole (LVPWd) and end-systole (LVPWs), and FS. The Teichholz formula was used to calculate EF. When the echocardiographic analyses were done, the animals were euthanized by decapitation [30].

### 4.8. Langendorff Protocol

After finishing echocardiographic analyses, animals were sacrificed by decapitation, blood samples were collected for further analyses, while via emergency thoracotomy, the hearts were quickly isolated, immersed in cold saline, and retrogradely perfused through the aorta in a Langendorff apparatus with Krebs–Henseleit solution. ((mmol/L): NaCl 118, KCl 4.7, CaCl_2_ × 2H_2_O 2.5, MgSO_4_ × 7H_2_O 1.7, NaHCO_3_ 25, KH_2_PO_4_ 1.2, glucose 11, pyruvate 2, equilibrated with 95% O_2_ plus 5% CO_2_, and warmed to 37 °C (pH 7.4)). Firstly, the left atrium was incised, followed by mitral valve separation in order to insert the transducer into the left ventricle. The following parameters of the myocardial function were continuously measured (at each perfusion pressure): dp/dt max, dp/dt min, SLVP, DLVP, and HR. CF was measured flowmetrically. In order to examine the heart function, after the stabilization period, the perfusion pressure was gradually decreased to 60 cm H_2_O, and then increased to 80, 100 and 120 cm H_2_O. Subsequently, the perfusion pressure was decreased to 40 cm H_2_O, and the same protocol was repeated, increasing the pressure from 40 to 120 cm H_2_O [31].

### 4.9. Redox Status Analysis

Parameters of oxidative stress were determined from the collected blood samples. Plasma samples were used to determine the concentration of TBARS, NO_2_^−^, O_2_^−^, and H_2_O_2_, while red blood cells lysate samples were used to determine the activities of SOD, CAT, and concentration of GSH. All markers were measured spectrophotometrically (Shimadzu UV-1800 spectrophotometer, Kjota, Japan) according to well-known methods described in detail in our previous research [21].

### 4.10. Inflammatory Status Analysis

Serum concentrations of IL-1β, IL-6, IL-22, IL-23, and IL-17A were determined in collected samples using commercially available ELISA kits. IL-1β, IL-6, IL-22, and IL-23 were measured with rat ELISA kits from FineTest (Wuhan Fine Biotech Co., Ltd., Wuhan, China), while IL-17A was quantified using a rat ELISA kit from Reed Biotech (Wuhan, China), as specified by the manufacturers. All assays were performed on microtiter plates pre-coated with cytokine-specific monoclonal antibodies. Standards and serum samples were added to the wells and incubated with biotin-labeled detection antibodies, followed by color development proportional to cytokine concentration. Absorbance was measured at 450 nm using an ELISA plate reader (UT-2100C, MCR Lab, Holon, Israel), and cytokine levels were expressed as pg/mL. The assay detection ranges were 31.25–2000 pg/mL for IL-1β, 62.5–4000 pg/mL for IL-6, 15.625–1000 pg/mL for IL-22, 15.625–1000 pg/mL for IL-23, and 15.63–1000 pg/mL for IL-17A [17].

### 4.11. Histopathological Analysis of the Heart

After completion of the Langendorff perfusion protocol, the hearts were excised, weighed, and longitudinally sectioned into left and right halves for further histopathological analysis. Cardiac tissues were fixed in 4% neutral-buffered paraformaldehyde, dehydrated through ascending concentrations of ethanol (70%, 96%, and 100%), cleared in xylene, embedded in paraffin, and sectioned into 5 μm-thick slices. Histopathological analysis included two staining methods: Picrosirius red staining for collagen detection and H&E staining for the assessment of cardiac morphology. Microscopic examination was performed using an Olympus BX51 (Olympus Corporation, Tokyo, Japan) light microscope. Morphometric analysis of cardiomyocytes, including measurements of cross-sectional area and diameter, was performed using calibrated Axiovision software 4.6 (Zeiss, White Plains, NY, USA). Collagen content was quantified using Image-Pro Plus software 7.0. (Media Cybernetics, Rockville, MD, USA), according to our previously published methodology [23].

### 4.12. Statistical Analysis

All statistical analyses of the obtained data were carried out using SPSS software (version 22.0; SPSS Inc., Chicago, IL, USA). Shapiro–Wilk and Kolmogorov–Smirnov tests were used to check the normality of data distribution. Differences among groups were evaluated by one-way ANOVA followed by the LSD and Tukey post hoc tests for multiple comparisons. In case of data not following a normal data distribution, the non-parametric Kruskal–Wallis test was used as an alternative. A *p*-value of less than 0.05 was considered statistically significant, and the results are expressed as mean ± standard deviation (SD).

## 5. Conclusions

Taken together, the results suggest that psoriasis promotes significant cardiovascular impairment characterized by reduced cardiac contractility and lusitropy, increased left ventricular systolic and diastolic pressures, enhanced oxidative stress and systemic inflammatory responses, and structural remodeling manifested as cardiomyocyte hypertrophy and fibrosis. Administration of MO extract effectively counteracted these alterations, leading to improved cardiac function, restoration of redox balance, suppression of inflammation, reduced myocardial hypertrophy and collagen accumulation, and improvement of cutaneous psoriatic manifestations. Based on this study`s findings, it can be concluded that MO ethanolic extract may be considered a valuable source of cardioprotective phytoconstituents that synergistically provide prevention of cardiac damage under psoriasis conditions. It represents a significant starting point for further mechanistic and translational research, aimed at evaluating the clinical potential of MO.

## Figures and Tables

**Figure 2:**
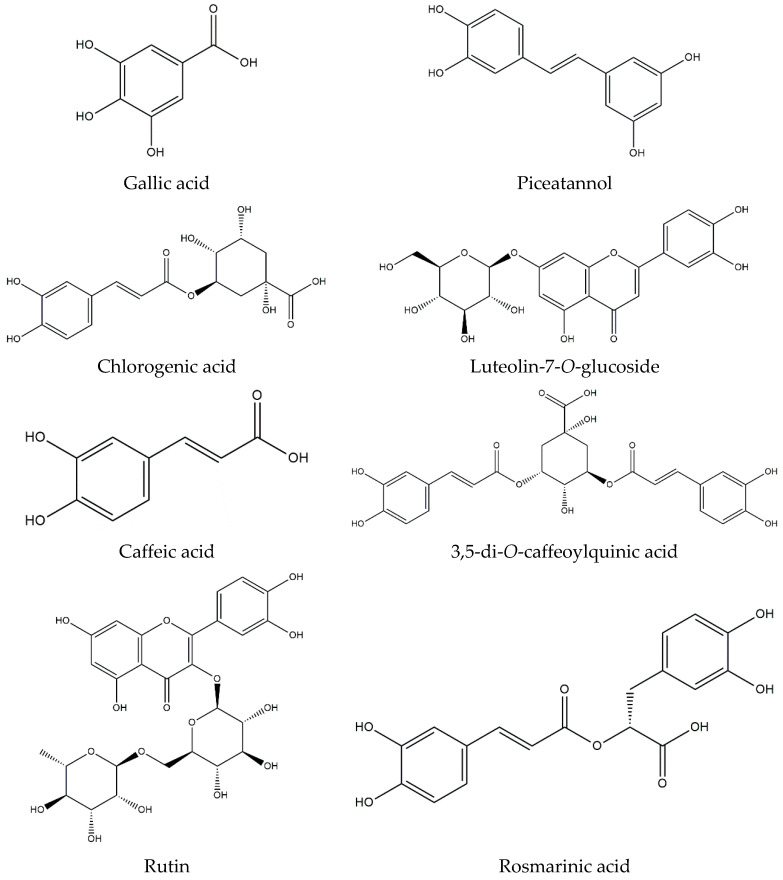
Chemical structures of the major phenolic constituents identified in the *Melissa officinalis* extract by HPLC-DAD analysis: gallic acid, chlorogenic acid, caffeic acid, rutin, piceatannol, luteolin-7-O-glucoside, 3,5-di-O-caffeoylquinic acid, and rosmarinic acid.

**Table 1:** Chemical characterization of the tested MO extract.

	Identified Compounds	Content mg/g DW of the Investigated Extract
1	Gallic acid	9.68 ± 0.21
2	Chlorogenic acid	3.76 ± 0.11
3	Caffeic acid	1.02 ± 0.09
4	Rutin	0.60 ± 0.02
5	Luteolin-7-O-glucoside	0.58 ± 0.02
6	Piceatanol	0.81 ± 0.05
7	3,5-di-O-caffeoylquinic acid	2.41 ± 0.10
8	Rosmarinic acid	57.53 ± 0.55

**Table 2:** IC_50_ values of antioxidant activity of the tested samples determined by DPPH and ABTS assays.

Sample	DPPH IC_50_ (µg/mL)	ABTS IC_50_ (µg/mL)
MO extract	78.14 ± 6.82a	88.63 ± 9.04a
AA	8.43 ± 0.73b	9.71 ± 0.85b
BHA	10.89 ± 0.97b	12.74 ± 1.06b
Trolox	4.86 ± 0.16b	6.29 ± 0.68b

MO—*Melissa officinalis*; АА—ascorbic acid; BHA—Butylated hydroxyanisole; Different letters indicate statistically significant differences (*p* < 0.05).

**Table 3:** Values of BW, HW, and index hypertrophy of heart (HW/BW ratio) after three weeks of treatment with MO.

Groups	BW	HW	HW/BW Ratio
CTRL	418.33 ± 23.19	1.46 ± 0.10	0.35 ± 0.01
MO	418.67 ± 29.08	1.46 ± 0.09	0.35 ± 0.00
PSORI	459.67 ± 8.08 ^a,b^	1.85 ± 0.10 ^a,b^	0.40 ± 0.01 ^a,b,c^
PSORI+MO	448.00 ± 7.95 ^a,b,c^	1.68 ± 0.06 ^a,b,c^	0.38 ± 0.01 ^a,b,c^

Results presented as mean value ± SD (n = 6). Statistical significance between groups is shown as a, b, c. a—denotes vs. CTRL; b—denotes vs. MO; c denotes vs. PSORI; CTRL (control), MO (*Melissa officinalis*), PSORI (psoriasis). BW—body weight; HW—heart weight.

**Table 4:** Average values of echocardiographic evaluation: IVSd—intraventricular septal wall thickness at end-diastole; LVIDd—left ventricle internal dimension at end-diastole; LVPWd—left ventricle posterior wall thickness at end-diastole; IVSs—intraventricular septal wall thickness at end-systole; LVIDs—left ventricle internal dimension at end-systole; LVPWs—left ventricle posterior wall thickness at end-systole; FS—fractional shortening percentage; EF—ejection fraction.

	CTRL	MO	PSORI	PSORI+MO
IVSd (cm)	0.133 ± 0.005	0.130 ± 0.010	0.117 ± 0.026	0.110 ± 0.008
LVIDd (cm)	0.837 ± 0.027	0.743 ± 0.048 ^a,c^	0.850 ± 0.080 ^b^	0.780 ± 0.003 ^a,c^
LVPWd (cm)	0.156 ± 0.003	0.130 ± 0.005	0.138 ± 0.049	0.124 ± 0.004 ^a^
IVSs (cm)	0.188 ± 0.012	0.195 ± 0.022	0.163 ± 0.055	0.189 ± 0.018
LVIDs (cm)	0.541 ± 0.007	0.390 ± 0.015 ^a^	0.566 ± 0.041 ^b^	0.438 ± 0.004 ^a,b,c^
LVPWs (cm)	0.233 ± 0.006	0.198 ± 0.018	0.157 ± 0.096 ^a^	0.196 ± 0.013
FS (%)	40.2 ± 0.916	47.33% ± 4.960 ^a^	32.03% ± 3.300 ^a,b^	43.93 ± 3.85 ^c^
EF (%)	74.9 ± 0.822	83.3 ± 4.479 ^a^	67.3 ± 4.648 ^a,b^	80.1 ± 3.96 ^a,c^

Results presented as mean value ± SD (n = 6). Statistical significance between groups is shown as a, b, c. a—denotes vs. CTRL; b—denotes vs. MO; c denotes vs. PSORI. CTRL—healthy non-treated rats; MO—healthy rats treated with MO extract; PSORI—rats with psoriasis and PSORI+MO—rats with psoriasis treated with MO extract.

## Data Availability

The original contributions presented in this study are included in the article and Supplementary Material. Further inquiries can be directed to the corresponding author.

## Supplementary Materials

The following supporting information can be downloaded at: https://www.mdpi.com/article/10.3390/plants15152342/s1, Figure S1: UV absorption spectrum of gallic acid identified in the *Melissa officinalis* extract by HPLC-DAD analysis; Figure S2: UV absorption spectrum of chlorogenic acid identified in the *Melissa officinalis* extract by HPLC-DAD analysis; Figure S3: UV absorption spectrum of caffeic acid identified in the *Melissa officinalis* extract by HPLC-DAD analysis; Figure S4: UV absorption spectrum of rutin identified in the *Melissa officinalis* extract by HPLC-DAD analysis; Figure S5: UV absorption spectrum of piceatannol identified in the *Melissa officinalis* extract by HPLC-DAD analysis; Figure S6: UV absorption spectrum of luteolin-7-O-glucoside identified in the *Melissa officinalis* extract by HPLC-DAD analysis; Figure S7: UV absorption spectrum of 3,5-di-O-caffeoylquinic acid identified in the *Melissa officinalis* extract by HPLC-DAD analysis; Figure S8: UV absorption spectrum of rosmarinic acid identified in the *Melissa officinalis* extract by HPLC-DAD analysis.

## Abbreviations

The following abbreviations are used in this manuscript:

MO	*Melissa officinalis*
TPC	Total phenolic content
FRAP	Ferric reducing antioxidant power
AA	Ascorbic acid
ABTS	2,2′-azino-bis-3-ethylbenzothiazoline-6-sulfonic
DPPH	2,2-diphenyl-1-picrylhydrazyl
BHA	Butylated hydroxyanisole
PASI	Psoriasis area and severity index
BW	Body weight
HW	Heart weight
CTRL	Control
LVIDd	LV internal dimension at end-diastole
FS	Fractional shortening
EF	Ejection fraction
LVIDs	LV internal dimension at end-systole
dp/dt max	Maximum rate of pressure development in the left ventricle
dp/dt min	Minimum rates of pressure development in the left ventricle
SLVP	Systolic left ventricular pressure
DLVP	Diastolic left ventricular pressure
HR	Heart rate
CF	Coronary flow
TBARS	Thiobarbituric acid reactive substances
SOD	Superoxide dismutase
GSH	Reduced glutathione
CAT	Catalase
CSA	Cross-sectional area
H/E	Hematoxylin/eosin
DAD	Diode array detector
IVSd	IVS wall thickness at end-diastole
IVSs	IVS wall thickness at end-systole
IVS	Inter-ventricular septum
LVPW	LV posterior wall
