# Supplementary material for: Melissa officinalis Mitigates Psoriasis-Associated Cardiac Remodeling Through Antioxidant and Anti-Inflammatory Mechanisms
Source: Plants (Basel). 2026 Jul 30;15(15):2342. doi: 10.3390/plants15152342 (PMC13468160; doi:10.3390/plants15152342)
Supplement: Supplementary file 1 [file plants-15-02342-s001.zip › plants-4429430-supplementary.pdf]

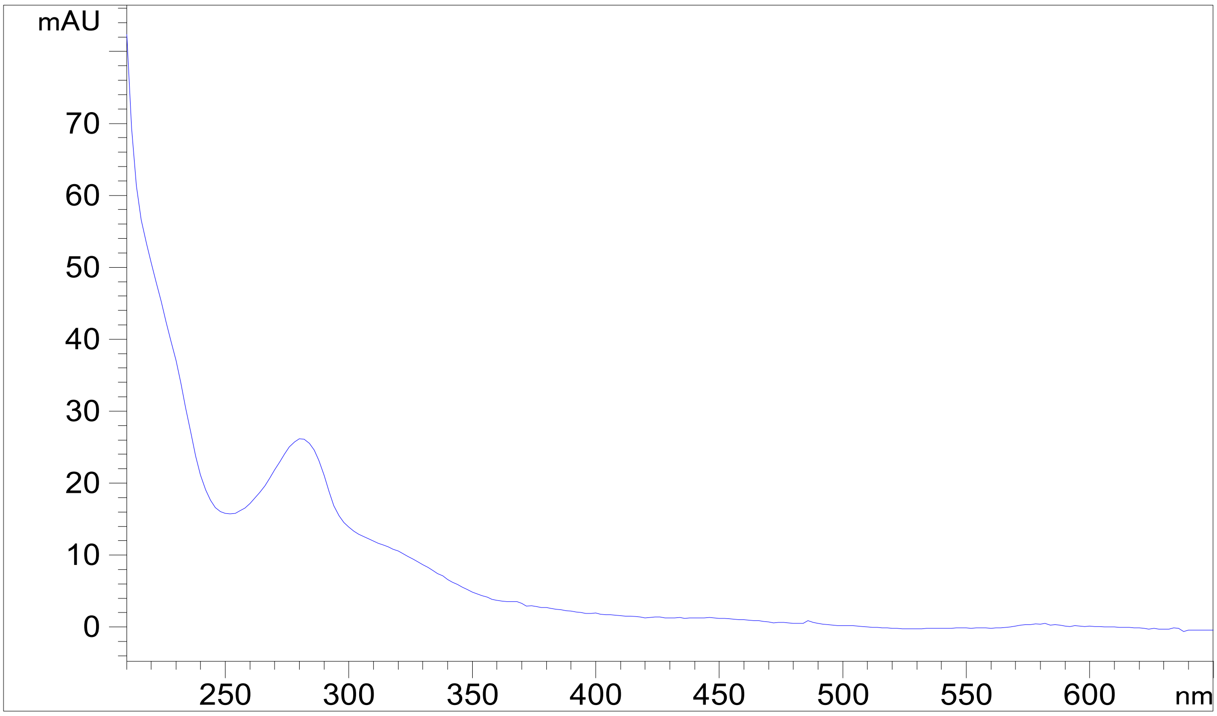

Figure S1. UV absorption spectrum of gallic acid identified in the *Melissa officinalis* extract by HPLC-DAD.

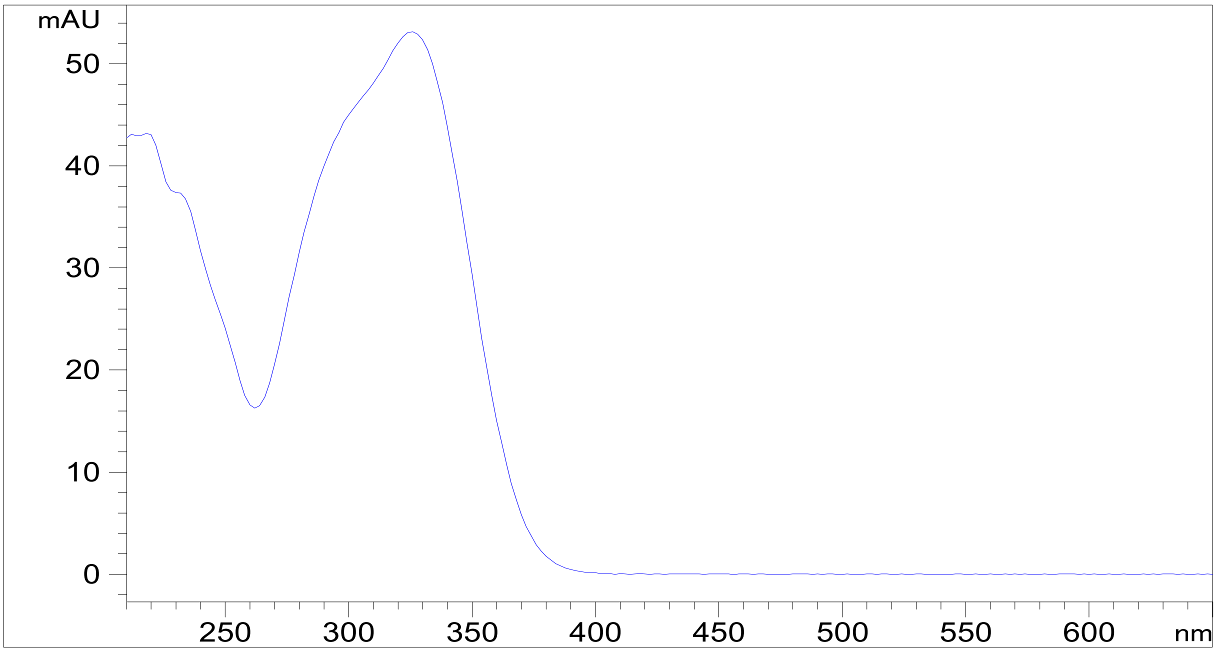

Figure S2. UV absorption spectrum of chlorogenic acid identified in the *Melissa officinalis* extract by HPLC-DAD.

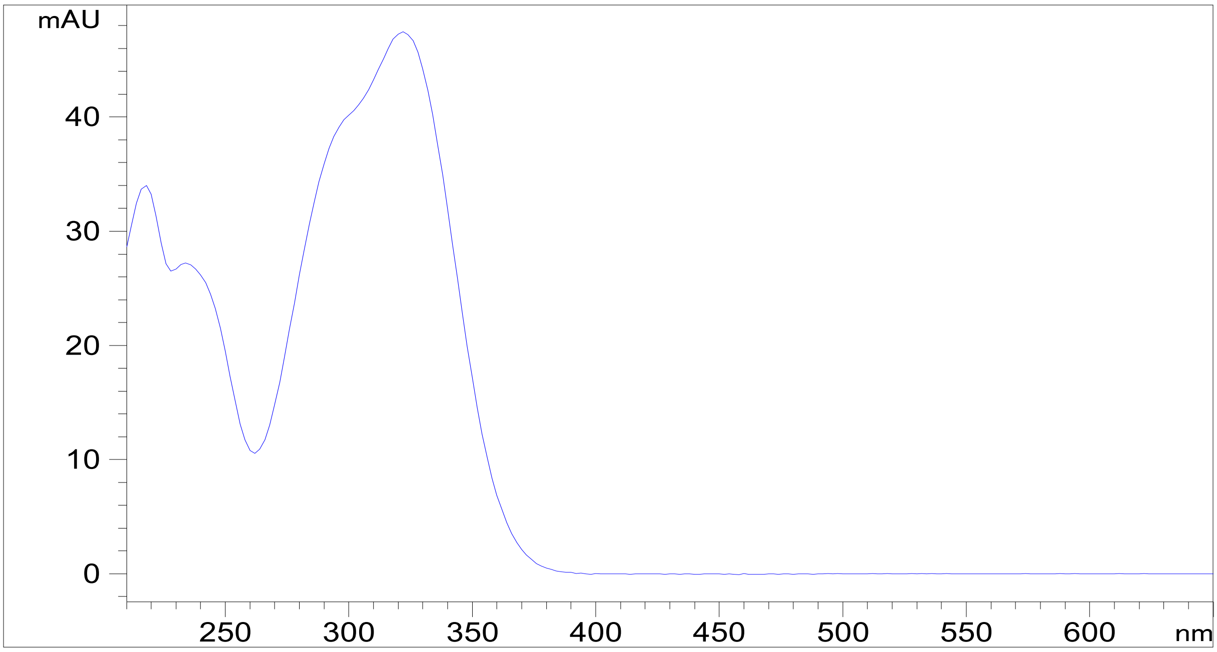

Figure S3. UV absorption spectrum of caffeic acid identified in the *Melissa officinalis* extract by HPLC-DAD.

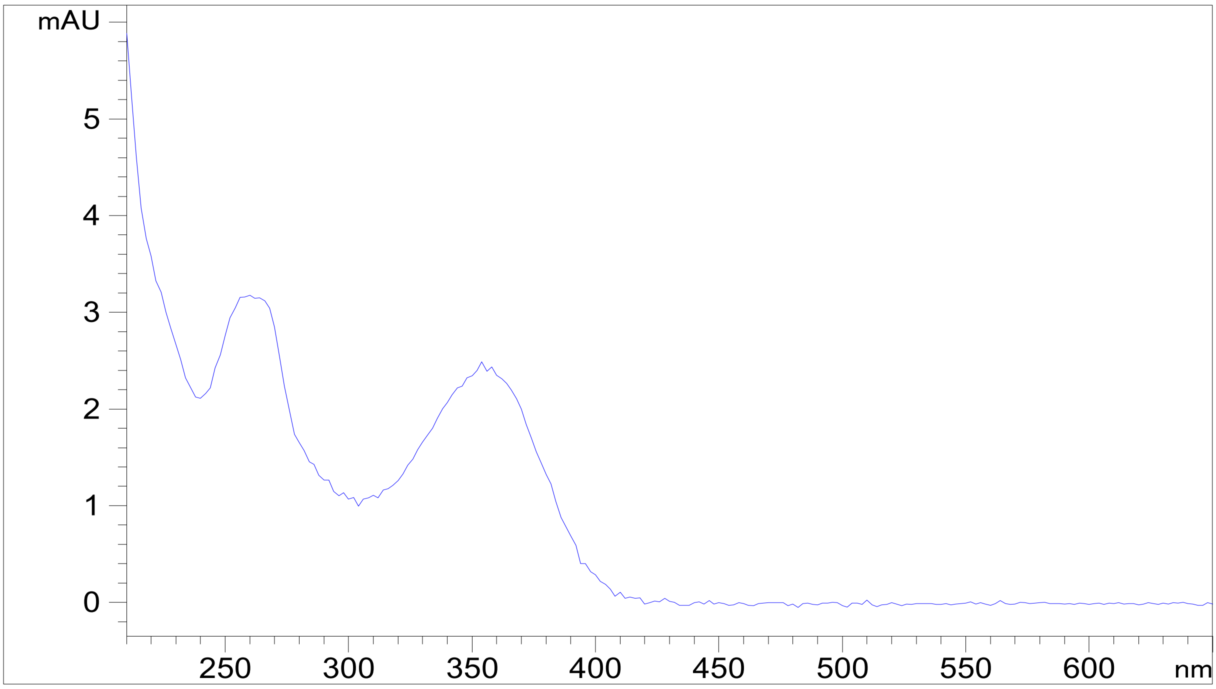

Figure S4. UV absorption spectrum of rutin identified in the *Melissa officinalis* extract by HPLC-DAD.

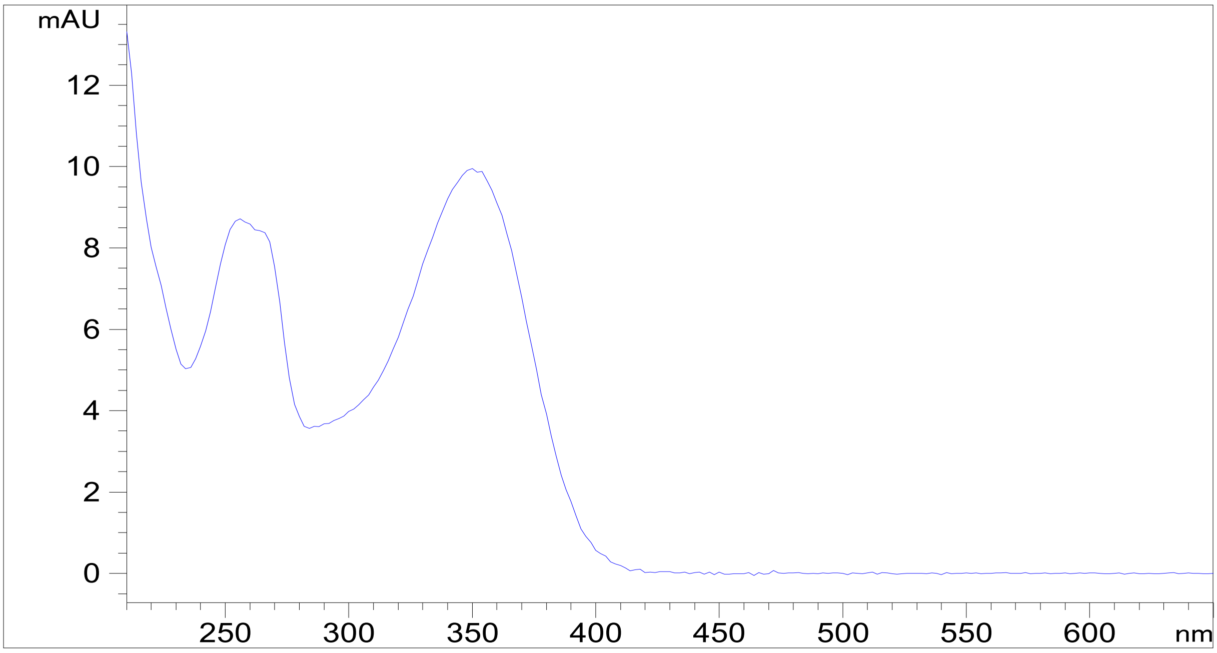

Figure S5. UV absorption spectrum of piceatannol identified in the *Melissa officinalis* extract by HPLC-DAD.

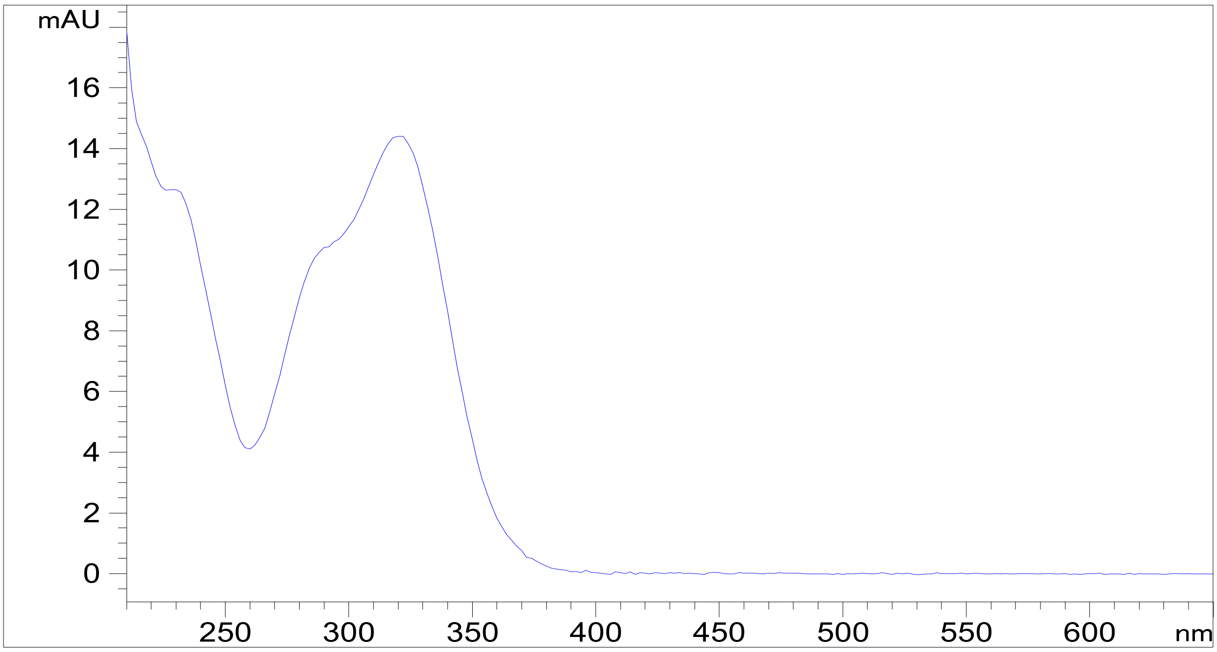

Figure S6. UV absorption spectrum of luteolin-7-O-glucoside identified in the *Melissa officinalis* extract by HPLC-DAD.

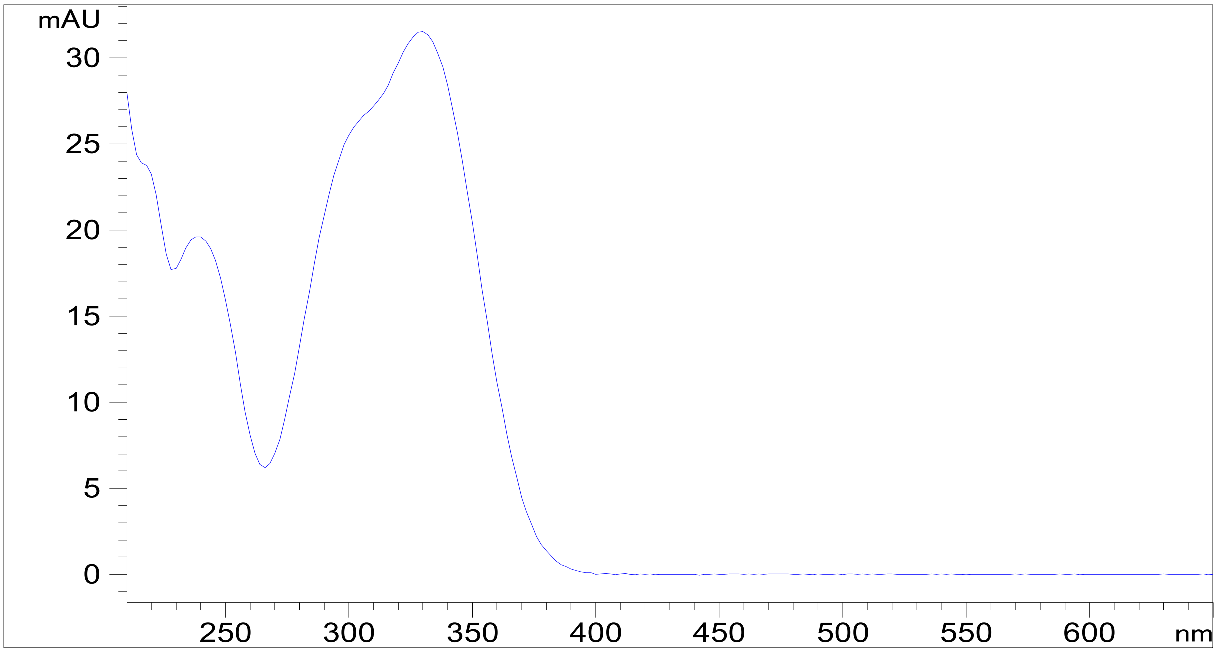

Figure S7. UV absorption spectrum of 3,5-di-O-caffeoylquinic acid identified in the *Melissa officinalis* extract by HPLC-DAD.

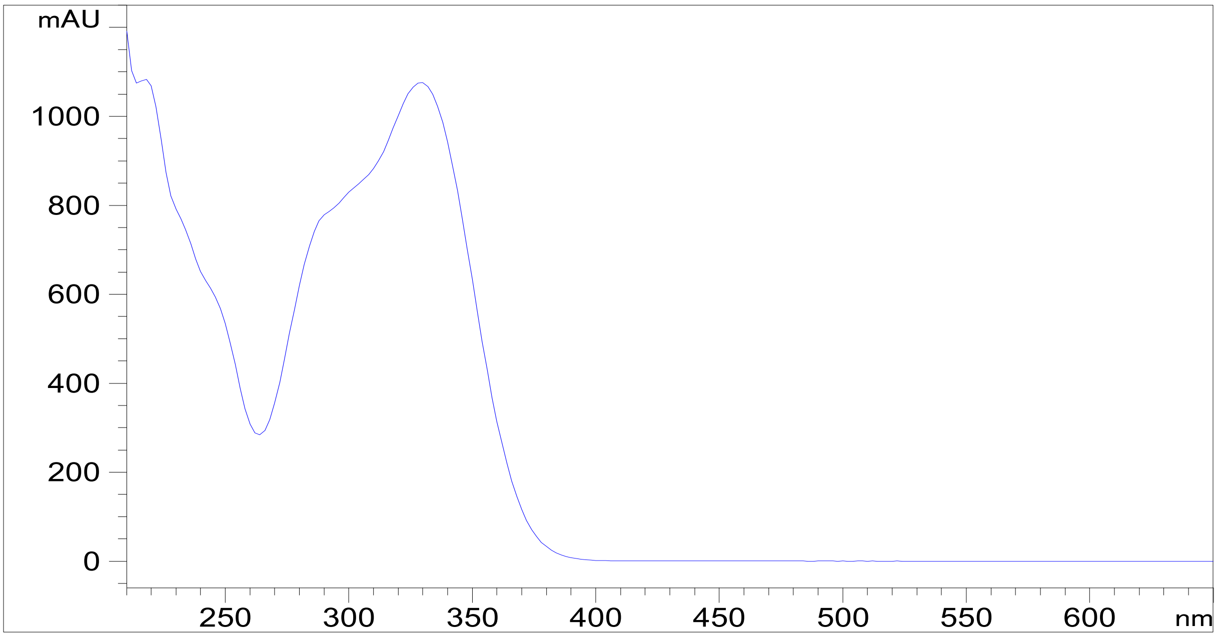

Figure S8. UV absorption spectrum of rosmarinic acid identified in the *Melissa officinalis* extract by HPLC-DAD.
